# Dose-response association of serum alanine aminotransferase levels with multimorbidity

**DOI:** 10.1038/s41598-019-44510-x

**Published:** 2019-05-30

**Authors:** Yuxia Zhang, Lugang Yu, Xiaoying Wang, Liqiang Qin, Yueping Shen, Chaofu Ke

**Affiliations:** 1 Department of Epidemiology and Biostatistics, School of Public Health, Medical College of Soochow University, 199 Renai Road, Suzhou, 215123 P.R. China; 2 Suzhou Industrial Park Centers for Disease Control and Prevention, Suzhou, 215021 Jiangsu, P.R. China; 3 College of Public Health, Sichuan University, Chengdu, 610041 P.R. China; 4 Medical Technology Department of Suzhou Vocational Health College, Suzhou, 215009 P.R. China; 5 Department of Nutrition and Food Hygiene, School of Public Health, Medical College of Soochow University, 199 Renai Road, Suzhou, 215123 P.R. China

**Keywords:** Biomarkers, Public health

## Abstract

Multimorbidity has posed a major challenge to health care systems worldwide, but little is known about its association with biological factors. This study represents the first one to examine the association of serum levels of alanine aminotransferase (ALT) with multimorbidity. The baseline category logistic regression model was used to estimate the odds ratio (OR) between ALT and multimorbidity, and the area under the receiver operating characteristic curve (AUC) was used to evaluate the classification utility of ALT. Serum ALT levels were associated, in a dose-response manner, with multimorbidity. Compared with the first quartile, the adjusted OR (95% confidence interval) of the fourth quartile for multimorbidity was 4.71 (3.56–6.23). In addition, the AUC value for distinguishing the multimorbidity group from the control group reached 0.7185. In conclusion, high levels of ALT were significantly correlated with multimorbidity and this association was independent of other potential risk factors. Serum ALT might be a useful marker for identifying individuals with multimorbidity.

## Introduction

With rapid population aging and the prevalence of chronic non-communicated diseases, coexistence of two or more chronic diseases, termed as multimorbidity^1^, has become a prominent problem worldwide^2^. Reports of its prevalence vary between 20~30% in the general population and between 55~98% among elderly adults^3^. It has been demonstrated that the impact of multimorbidity represents more than the sum of the individual effects from these conditions^4^. Multimorbidity not only endangers individual health but also poses a great challenge to the country’s health-care system. Researches show that each additional chronic disease reduces life expectancy by an average of 1.8 years^5^, and more than 2/3 of health resources are used by multimorbidity patients^6^. Therefore, taking positive measures to respond and investigating the biological factors of multimorbidity are of great significance for disease management.

Alanine aminotransferase (ALT) mainly exists in the cytoplasm of the liver, and it is regarded as the most sensitive indicator for the detection of liver dysfunction^7^. However, large amounts of epidemiological researches have also provided evidences for a link between ALT and overall health or all-cause mortality^8–10^. In addition, ALT has been associated with increased risks of metabolic syndrome, diabetes mellitus and cardiovascular disease^10–14^. To date, previous studies have mainly focused on the association of ALT with a specific disease, but few studies have explored the relationship between ALT and multimorbidity. In this study, we investigated the association between the serum ALT levels and multimorbidity in a southeast Chinese population.

## Methods

### Study population

The study population was from those enrolled for physical examination from July 2013 to November 2014 in the in Suzhou Industrial Park (Suzhou, China). All participants completed a health questionnaire and physical examination under a standardized protocol. The exclusion criteria include the individuals who had an incomplete records of ALT and chronic diseases. A total of 5890 participants (2613 men and 3277 women) with complete data were included in our final analysis. All participants signed the informed consent, and the protocol was approved by the Ethics Committee of Soochow University. All methods were performed in accordance with relevant guidelines and regulations.

### Data collection and measurements

Designed questionnaires were used to collect the information about demographics, socioeconomics, lifestyle behaviors and healthcare characteristics. Medical history was collected by trained doctors through face-to-face interviews. Blood pressure (BP) was measured in a seated position three times consecutively at 1 minute intervals using a manual mercury sphygmomanometer. The overnight 10–12 h fasting serum samples were collected and provided for measurement of the total cholesterol (TC), triglycerides (TG), alanine aminotransferase (ALT), aspartate aminotransferase (AST), high density lipoprotein cholesterol (HDLC), low density lipoprotein  cholesterol (LDLC), glycosylated hemoglobin (GHB), and fasting blood glucose (FBG) which were analyzed using an Olympus AU640 auto analyzer (Olympus, Kobe, Japan). Standard laboratory assay methods and quality control methods were conducted.

### Definition of multimorbidity

Chronic diseases included hypertension, diabetes, dyslipidemia, coronary heart disease, stroke, cancer and chronic nephropathy. These diseases were diagnosed through face-to-face interviews, clinical examinations, and laboratory tests. Besides the self-reported cases, hypertension, dyslipidemia and diabetes were diagnosed using the following criteria as well. Hypertension was determined by systolic BP ≥140 mmHg or diastolic BP ≥90 mmHg. Diabetes was determined by FBG ≥7.0 mmol/L or GHB ≥6.5%. Dyslipidemia was defined as TC ≥6.22 mmol/L, or TG ≥2.26 mmol/L, or HDLC <1.04 mmol/L, or LDLC ≥4.14 mmol/L. According to the definition by WHO, multimorbidity refers to patients suffering from two or more chronic diseases at the same time. In line with other studies, all chronic diseases were weighted equally^15,16^.

### Statistical analysis

For continuous variables with non-normal distribution, they were described as median (inter-quartile range, IQR). Categorical variables were presented as frequency (percentage). Continuous variables, including age, BMI, SBP, DBP, TC, TG, LDLC, HDLC, GHB, FBG, AST and ALT, were compared among the groups of control, single disease and multimorbidity by the Kruskal-Wallis test. Categorical variables, including gender, smoking, drinking, exercise, income, education and marriage, were compared among the groups of control, single disease and multimorbidity by the chi-square test. Multiple comparisons between groups were performed by Bonferroni adjustment and the significance level was set as α′ = 0.05/3 = 0.017. According to the 25th percentile, 50th percentile and 75th percentile of ALT levels among the participants, we divided the population into four groups: the bottom quartile group (Q1) with ALT ≤13.05 U/L; the second quartile group (Q2) with 13.05 U/L < ALT ≤18.00 U/L; the third quartile group (Q3) with 18.00 U/L < ALT ≤25.00 U/L and the highest quartile group (Q4) with ALT >25.00 U/L. Unadjusted and adjusted odds ratios (ORs) and 95% confidence intervals (CIs) of ALT quartiles were calculated by the baseline category logistic regression model. The dependent variable in the model was the disease status (control, single disease and multimorbidity), and the control group was used as the reference. The explanatory variables in the model were ALT and potential confounders (e.g. gender, educational level, income, marital status, smoking, drinking, BMI, exercise and chronic liver disease). To investigate the classification potential of ALT for multimorbidity, receiver operating characteristic curve (ROC) analysis and calculation of area under ROC (AUC) for single disease *vs*. control, multimorbidity *vs*. non-multimorbidity and multimorbidity *vs*. control were implemented by the “pROC” package in the R platform^17^. In pROC, the ROC curves were empirical curves in the sensitivity and specificity space, and AUC values were computed with trapezoids^17^. In all analyses, the significance level was set at α = 0.05. All the statistical analyses were performed by R 3.3.3.

## Results

### Subject characteristics

According to the number of chronic diseases, study subjects were classified into three groups, namely the control group, the single disease group and the multimorbidity group. The proportions of the three groups were 31.37%, 40.07% and 28.56% respectively. Gender, age, BMI, smoking, drinking, education, marriage status, SBP, DBP, TC, TG, LDLC, HDLC, GHB, FBG, AST and ALT were significantly different among the three groups, with the exception of exercise (Table 1). It can be seen that the median age of multimorbidity was 58 years old, significantly higher than the other groups. The prevalence of multimorbidity in men (31.84%) was significantly higher than that in women (25.94%). Furthermore, the subjects in multimorbidity group had significantly higher levels of BMI, SBP, DBP, TC, TG, LDLC, GHB, and FBG than in another two groups. In addition, higher prevalence of multimorbidity was found in individuals with unfavorable marital status and unhealthy lifestyle factors such as smoking and drinking.

**Table 1:** The characteristics of the study population.

Characteristics	Control (n = 1848)	Single disease (n = 2360)	Multimorbidity (n = 1682)	*P* value
Age (years)	51(45,60)	56(48,63)	58(48,66)	<0.0001
Gender				<0.0001
Men	680(26.02)	1101(42.14)	832(31.84)	
Women	1168(35.64)	1259(38.42)	850(25.94)	
Education				<0.0001
Lower	1090(28.99)	1533(40.77)	1137(30.24)	
Higher	747(35.52)	819(38.94)	537(25.53)	
Marriage				<0.0001
Cohabitation	1772(31.95)	2225(40.12)	1549(27.93)	
Living alone	46(17.76)	102(39.38)	111(42.86)	
Monthly income (¥)				<0.0001
≤1,000	448(26.03)	656(38.12)	617(35.85)	
1,000–3,000	916(31.07)	1195(40.54)	837(28.39)	
≥3,000	466(40.10)	493(42.43)	203(17.47)	
Smoking				0.0007
Yes	476(27.87)	705(41.28)	527(30.85)	
No	1340(32.75)	1626(39.74)	1126(27.52)	
Drinking				<0.0001
Yes	282(24.80)	502(44.15)	353(31.05)	
No	1550(33.02)	1835(39.09)	1309(27.89)	
Exercise				0.7485
Yes	361(31.98)	447(39.59)	321(28.43)	
No	1294(31.33)	1687(40.85)	1149(27.82)	
BMI	22.61(20.82,24.51)	24.01(22.04,26.03)	24.93(23.01,27.22)	<0.0001
SBP (mmHg)	116.00(108.00,124.00)	132.00(119.00,143.00)	138.00(128.00,149.00)	<0.0001
DBP (mmHg)	73.00(67.00,78.00)	83.00(75.00,91.00)	85.00(79.00,92.00)	<0.0001
TC (mmol/L)	4.67(4.16,5.24)	4.78(4.18,5.47)	5.03(4.27,5.84)	<0.0001
TG (mmol/L)	1.03(0.77,1.36)	1.28(0.93,1.85)	2.20(1.36,3.44)	<0.0001
LDLC (mmol/L)	2.59(2.18,3.05)	2.72(2.29,3.22)	2.78(2.17,3.41)	<0.0001
HDLC (mmol/L)	1.42(1.23,1.61)	1.34(1.08,1.57)	1.06(0.92,1.32)	<0.0001
GHB (%)	5.80(5.60,6.00)	5.90(5.70,6.10)	6.40(5.80,60.90)	<0.0001
FBG (mmol/L)	5.48(5.07,5.90)	5.59(5.18,6.04)	5.91(5.55,7.12)	<0.0001
AST (mmol/L)	22.00(19.00,26.92)	23.00(19.00,28.01)	22.53(19.00,27.83)	<0.0001
ALT (mmol/L)	16.00(12.00,20.73)	18.00(14.00,24.00)	22.00(17.00,31.00)	<0.0001

BMI, body mass index; SBP, systolic blood pressure; DBP, diastolic blood pressure; TC, total cholesterol; TG, triglyceride; LDLC, low-density lipoprotein cholesterol; HDLC, high-density lipoprotein cholesterol; GHB, glycosylated hemoglobin; FBG, fasting blood glucose; AST, aspartate aminotransferase; ALT, alanine aminotransferase.

### Distribution of ALT within different statuses

**Figure 1:**
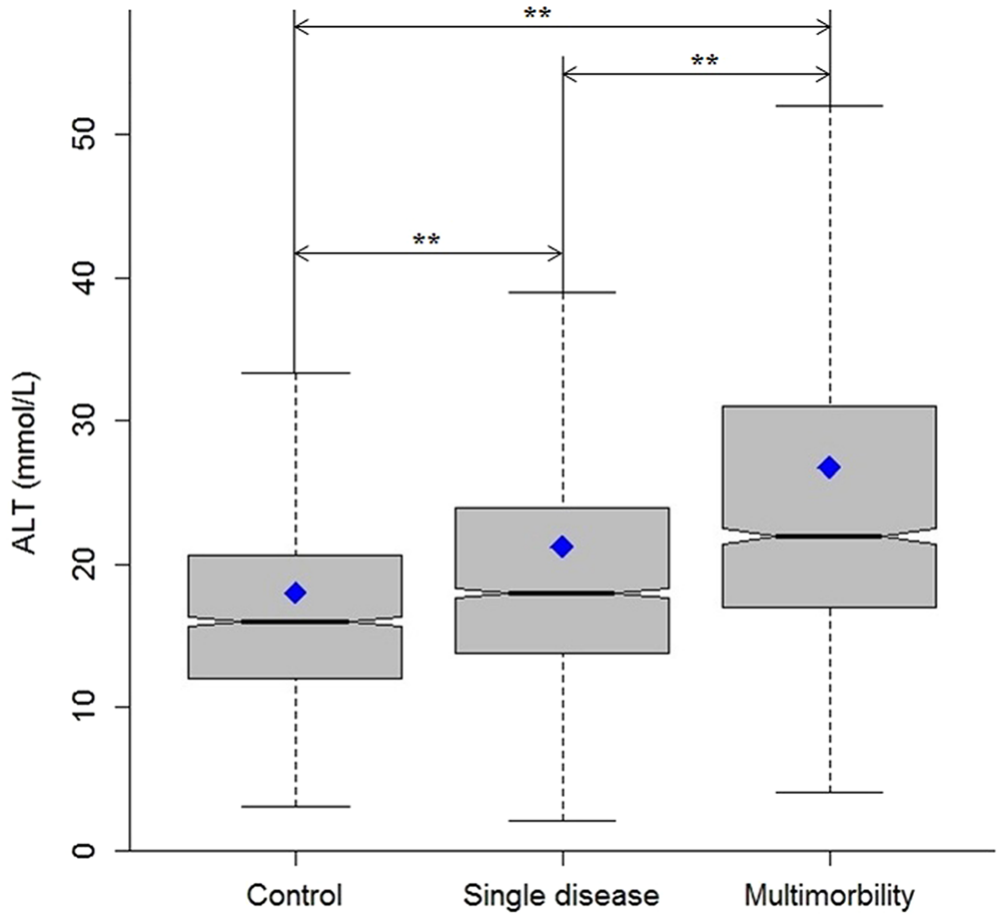
The box plots of ALT levels among the groups of control, single disease and multimorbidity (**P < 0.0001; the blue diamond in the box plot indicates the mean of ALT in each group).

Levels of ALT showed significant difference among the control group, the single disease group and the multimorbidity group after using the Kruskal-Wallis test (*P* < 0.0001). Moreover, increasing trends of ALT from the control to the single disease and multimorbidity groups were also obviously observed. The median levels of ALT were 16, 18 and 22 mmol/L in the three groups (Fig. 1). Further multiple comparisons between groups also showed statistical significance at the Bonferroni-adjusted significance level.

### Logistic regression analysis for the association between ALT and multimorbidity

Higher ALT levels were found to be associated, in a dose-response manner, with multimorbidity. Compared with the bottom quartile of ALT, the unadjusted OR (95% CI) of the single disease patients increased from 1.44 (1.23–1.69) for the second quartile group to 2.23 (1.86–2.68) for the highest quartile group (*P* for trend < 0.0001). By contrast, the unadjusted OR (95% CI) of the multimorbidity patients increased from 2.01 (1.64–2.46) for the second quartile group to 7.61 (6.17–9.38) for the highest quartile group (*P* for trend < 0.0001). The ORs were attenuated with the adding of covariates, especially for the single disease group, but still remained statistically significant. As compared with the first quartile, adjusted ORs (95% CIs) for single disease were as follows: second quartile, 1.27(1.04–1.55); third quartile, 1.49 (1.19–1.87), and fourth quartile, 1.55 (1.22–1.98). Adjusted ORs (95% CIs) for multimorbidity were as follows: second quartile, 1.72 (1.32–2.24); third quartile, 3.31 (2.52–4.34), and fourth quartile, 4.71 (3.56–6.23). Remarkably, both unadjusted and adjusted OR values of the multimorbidity group were much bigger than those of the single disease group under the same conditions (Table 2).

**Table 2:** Baseline category logistic regression analysis for the association between ALT and single disease and multimorbidity compared to the control group.

	Unadjusted		Model 1		Model 2	
	OR	95%CI	OR	95%CI	OR	95%CI
Single disease						
Q1	1	—	1	—	1	—
Q2	1.44	(1.23–1.69)	1.31	(1.12–1.55)	1.27	(1.04–1.55)
Q3	1.85	(1.55–2.19)	1.64	(1.37–1.96)	1.49	(1.19–1.87)
Q4	2.23	(1.86–2.68)	2.00	(1.65–2.42)	1.55	(1.22–1.98)
*P* trend	<0.0001		<0.0001		0.0179	
Multimorbidity						
Q1	1	—	1	—	1	—
Q2	2.01	(1.64–2.46)	1.79	(1.45–2.21)	1.72	(1.32–2.24)
Q3	4.19	(3.41–5.15)	3.42	(2.76–4.24)	3.31	(2.52–4.34)
Q4	7.61	(6.17–9.38)	6.71	(5.39–8.35)	4.71	(3.56–6.23)
*P* trend	<0.0001		<0.0001		<0.0001	

Model 1: adjusted for age, gender, educational level, income and marital status.

Model 2: adjusted for age, gender, educational level, income, marital status, smoking, drinking, BMI, exercise and chronic liver disease.

OR, odds ratio; CI, confidence interval.

### Classification potential of ALT for multimorbidity

**Figure 2:**
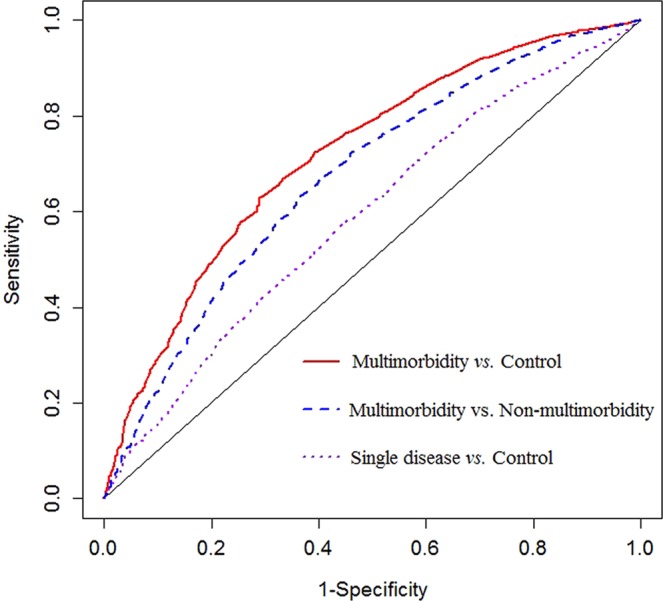
The potential of ALT for classifying multimorbidity.

The capacities of ALT for discriminating multimorbidity from single disease or control were further evaluated. As can be seen, the AUC value for distinguishing the single disease group from the control group was 0.5891. By contrast, the AUC values for discriminating the multimorbidity group from the non-multimorbidity group and from the control group have reached 0.6721 and 0.7185, respectively (Fig. 2).

## Discussion

Our results demonstrated that ALT was associated with multimorbidity and this association was independent of other related risk factors. The participants with multimorbidity were presented with significantly higher ALT than those who had only one single disease or none of the above diseases. In addition, ALT was found to be a potential biomarker for classification of multimorbidity.

Multimorbidity, as a growing issue, poses a major challenge to health care systems throughout the world^18^. In recent years, more and more scholars have been investigating the influential factors of multimorbidity from different aspects. Our findings were consistent with previous studies that older persons, persons who smoke or drink, and persons from low social classes were more likely to be affected by multimorbidity^3,19^. However, the phenomenon that higher prevalence of multimorbidity was seen in men than in women in our study was inconsistent with other studies^3,16,19^. This is probably due to the fact that the average age of the men in this survey was higher than that of the women.

To the best of our knowledge, no published researches have investigated the relationship between ALT and multimorbidity. Even so, there were a number of literatures on the relationship between ALT and a specific disease. Previous cohort studies have demonstrated that even within the physiological range, a high level of ALT was an independent risk factor of type 2 diabetes mellitus, impaired fasting glucose, cardiovascular diseases and metabolic syndrome^12,20,21^. Moreover, a recent meta-analysis found the positive association between ALT and cancer in Asian populations^22^. Our results might indicate that ALT could be one possible link among these diseases.

The biological mechanisms that underlie the associations between ALT and these diseases have been incompletely elucidated. Several potential mechanisms may be proposed. Firstly, large amounts of experimental and epidemiological evidences have supported the close link for ALT and insulin resistance^23–25^. Insulin resistance, a hallmark of type 2 diabetes mellitus, could be responsible for a clustering of various metabolic disorders, such as hypertension, dyslipidemia and atherosclerotic cardiovascular diseases^26–28^. Secondly, studies have shown that elevated ALT may be an indicator of chronic inflammation^29,30^. As an integral part of the chronic inflammatory response, oxidative stress could cause damage to a series of bioactive molecules (e.g. DNA, lipids, proteins, etc.), which may in turn result in the development and progression of cardiovascular diseases and cancer^31,32^. In addition, inflammation and oxidative stress could also damage the insulin signals in the liver, and may damage the blood vessels to cause atherosclerosis^33^. At last, ALT levels might reflect the activity of hormone synthesis in the liver, and poor liver function is expected to increase the risk of hormone-regulated cancer^22^. Taken together, several regulatory pathways, including insulin resistance, chronic inflammatory responses and hepatic hormone synthesis, may underlie the association of ALT with multimorbidity.

There are several limitations in the present study. First, the cross-sectional design prevented us from establishing a causal relationship, and this deserves to be investigated in the future prospective researches. Second, the inclusion manner of chronic diseases in this study, mainly through self-reporting, may underestimate the prevalence and effects of the co-existing diseases. Third, this study gave an equal weight to all diseases. It may have been expected that severe diseases (e.g. cancer, stroke, etc.) were endowed with higher weights than slight diseases (e.g. hypertension, dyslipidemia, etc.). However, we followed the most commonly used international definition of multimorbidity^1,15^. Distributing different weights to different diseases in multimorbidity remains to be further investigated in the future.

## Conclusions

Elevated serum ALT levels were independently associated, in a dose-response manner, with multimorbidity. Serum ALT might serve as a useful marker for identifying individuals with multimorbidity.
